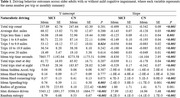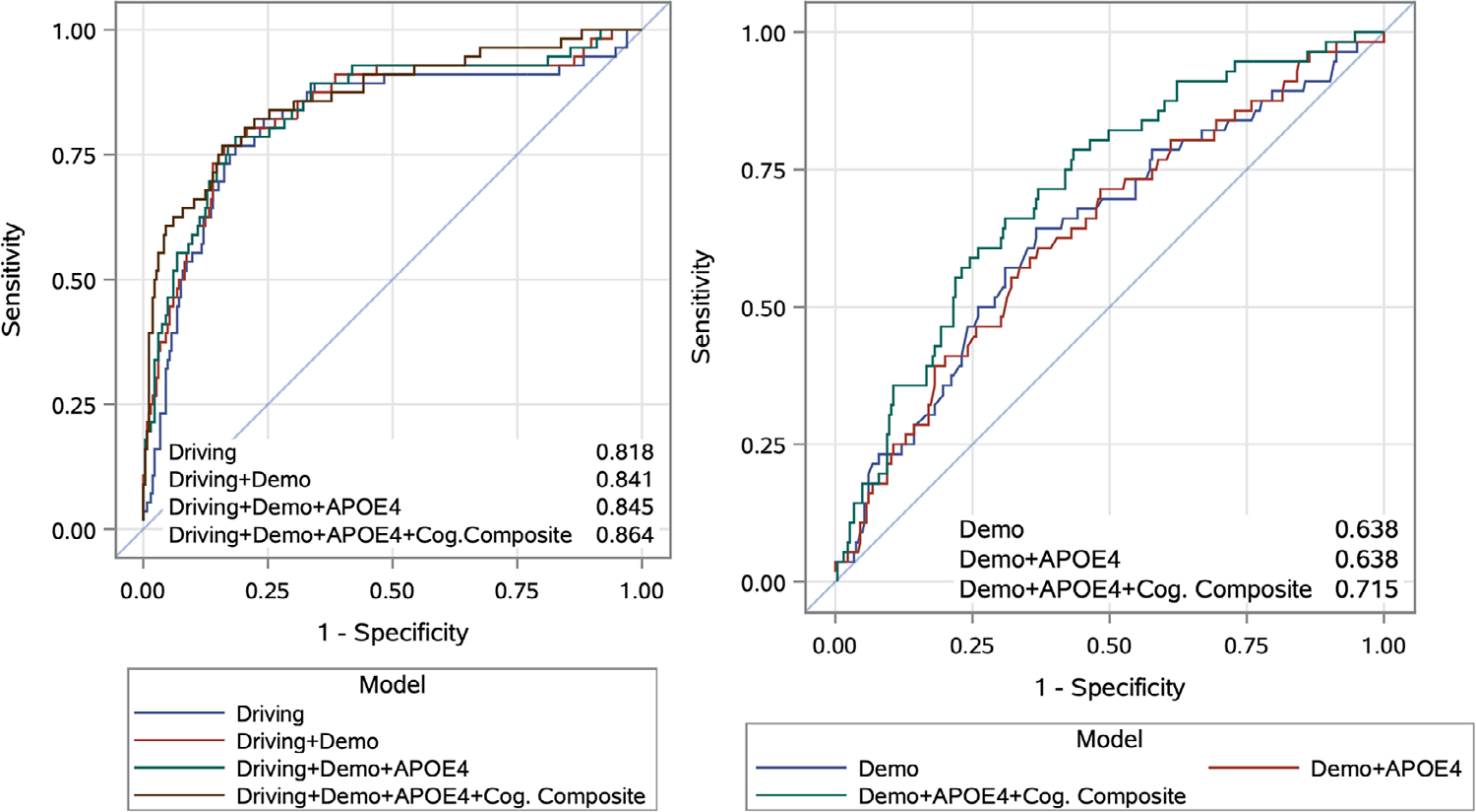# Early Detours: Daily Driving Behavior Predicts Mild Cognitive Decline in Older Adults

**DOI:** 10.1002/alz70861_108570

**Published:** 2025-12-23

**Authors:** Ling Chen, David B. Carr, Ramkrishna Kumar Singh, Semere Bekena, Yiqi Zhu, Kaylin Taylor, Jean‐Francois Trani, Ganesh M. Babulal

**Affiliations:** ^1^ Washington University in St. Louis, St. Louis, MO USA; ^2^ Washington University School of Medicine, St. Louis, MO USA; ^3^ Washington University School of Medicine, Saint Louis, MO USA; ^4^ Washington University, St. Louis, MO USA

## Abstract

**Background:**

Driving is a complex instrumental activity of daily living that supports independence in older adults but is adversely affected by age‐related cognitive decline. Mild cognitive impairment (MCI), a prodromal stage of dementia, increases crash risk, yet screening for fitness‐to‐drive remains critically underexamined. Naturalistic driving data collected via in‐vehicle dataloggers offers an unobtrusive and continuous method to detect early functional changes in older drivers at risk for Alzheimer’s disease and related dementias.

**Methods:**

Participants (*n* =321; MCI=56, CN=265) were enrolled in The DRIVES Project, a longitudinal cohort study at Washington University School of Medicine. They were required to be ≥65 years old, drive a non‐adapted vehicle at least weekly, and agree to complete annual clinical and neuropsychological assessments. Driving behavior was continuously recorded via an in‐vehicle GPS‐enabled datalogger over 40 months. Clinical diagnoses were determined using the Clinical Dementia Rating (CDR) and neuropsychological testing. Driving metrics—trip frequency, distance, time, spatial mobility, and risky behaviors—were aggregated monthly. Linear mixed‐effects models assessed group differences over time. Receiver operating curve analyses evaluated the driving metrics’ discriminatory ability to identify MCI.

**Results:**

While baseline driving behaviors between MCI and CN groups were largely similar, longitudinal analyses revealed significant divergence. MCI participants exhibited steeper declines in trip frequency (short‐, medium‐, and long‐distances), daytime driving, and trip duration (*p* <0.001) (Table 1). They also showed faster reductions in spatial mobility measures, including maximum distance driven, radius of gyration, and destination entropy (*p* <0.001), suggesting declining routines and lifespace. MCI drivers had fewer speeding events and slightly more hard cornering over time. ROC analyses demonstrated that driving features alone distinguished MCI from CN with AUC=0.82 (95% CI: 0.75–0.89). Adding demographics, APOE ε4 status, and cognitive composite improved classification to AUC=0.86 (95% CI: 0.81–0.92) (Figure 1).

**Conclusions:**

Older drivers with MCI show measurable and progressive changes in daily driving behavior, particularly in frequency, mobility, and navigational variability. Datalogger‐derived metrics can reliably discriminate MCI and serve as passive digital biomarkers for early detection of MCI. These findings support integrating real‐world driving data into clinical assessments to enhance early identification and personalized interventions for at‐risk older adults.